# Nucleic Acid Amplification Testing and Sequencing Combined with Acid-Fast Staining in Needle Biopsy Lung Tissues for the Diagnosis of Smear-Negative Pulmonary Tuberculosis

**DOI:** 10.1371/journal.pone.0167342

**Published:** 2016-12-02

**Authors:** Faming Jiang, Weiwei Huang, Ye Wang, Panwen Tian, Xuerong Chen, Zongan Liang

**Affiliations:** Department of Respiratory and Critical Care Medicine, West China Medical School and West China Hospital, Sichuan University, Chengdu, Sichuan, China; Indian Institute of Technology Delhi, INDIA

## Abstract

**Background:**

Smear-negative pulmonary tuberculosis (PTB) is common and difficult to diagnose. In this study, we investigated the diagnostic value of nucleic acid amplification testing and sequencing combined with acid-fast bacteria (AFB) staining of needle biopsy lung tissues for patients with suspected smear-negative PTB.

**Methods:**

Patients with suspected smear-negative PTB who underwent percutaneous transthoracic needle biopsy between May 1, 2012, and June 30, 2015, were enrolled in this retrospective study. Patients with AFB in sputum smears were excluded. All lung biopsy specimens were fixed in formalin, embedded in paraffin, and subjected to acid-fast staining and tuberculous polymerase chain reaction (TB-PCR). For patients with positive AFB and negative TB-PCR results in lung tissues, probe assays and 16S rRNA sequencing were used for identification of nontuberculous mycobacteria (NTM). The sensitivity, specificity, positive predictive value (PPV), negative predictive value (NPV), and diagnostic accuracy of PCR and AFB staining were calculated separately and in combination.

**Results:**

Among the 220 eligible patients, 133 were diagnosed with TB (men/women: 76/57; age range: 17–80 years, confirmed TB: 9, probable TB: 124). Forty-eight patients who were diagnosed with other specific diseases were assigned as negative controls, and 39 patients with indeterminate final diagnosis were excluded from statistical analysis. The sensitivity, specificity, PPV, NPV, and accuracy of histological AFB (HAFB) for the diagnosis of smear-negative were 61.7% (82/133), 100% (48/48), 100% (82/82), 48.5% (48/181), and 71.8% (130/181), respectively. The sensitivity, specificity, PPV, and NPV of histological PCR were 89.5% (119/133), 95.8% (46/48), 98.3% (119/121), and 76.7% (46/60), respectively, demonstrating that histological PCR had significantly higher accuracy (91.2% [165/181]) than histological acid-fast staining (71.8% [130/181]), *P* < 0.001. Parallel testing of histological AFB staining and PCR showed the sensitivity, specificity, PPV, NPV, and accuracy to be 94.0% (125/133), 95.8% (46/48), 98.4% (125/127), 85.2% (46/54), and 94.5% (171/181), respectively. Among patients with positive AFB and negative PCR results in lung tissue specimens, two were diagnosed with NTM infections (*Mycobacterium avium-intracellulare* complex and *Mycobacterium kansasii*).

**Conclusion:**

Nucleic acid amplification testing combined with acid-fast staining in lung biopsy tissues can lead to early and accurate diagnosis in patients with smear-negative pulmonary tuberculosis. For patients with positive histological AFB and negative tuberculous PCR results in lung tissue, NTM infection should be suspected and could be identified by specific probe assays or 16S rRNA sequencing.

## Introduction

Pulmonary tuberculosis (PTB) is a major public health problem worldwide. Globally, of the 5.2 million patients with new or relapsed PTB diagnosed in 2014, 3.0 million (58%) were bacteriologically confirmed, and the remaining 42% were diagnosed on the basis of clinical suspicion [1]. In China, 4.99 million patients with active PTB were diagnosed in 2010, of whom only 0.72 million were smear-positive (thus, approximately 85.6% of patients with PTB were smear-negative) [2]. Without rapid acid-fast bacteria (AFB) staining evidence, smear-negative PTB is often difficult to identify and may require mycobacterial culture and pathological or molecular diagnostics.

Sputum culture is more sensitive than sputum AFB smears and can be used to identify the species of mycobacteria. Moreover, drug-susceptibility testing (DST) based on sputum is helpful for the treatment of drug-resistant mycobacteria infections. However, mycobacterial culture is time-consuming (requiring 2–6 weeks for results) [3], and the results are often invariably delayed because of the slow growth of mycobacteria [4]. Earlier laboratory confirmation of TB can lead to earlier treatment initiation, improved patient outcomes, increased opportunities to interrupt transmission, and more effective public health interventions [5, 6]. Moreover, AFB smears or mycobacterial cultures of respiratory specimens cannot be performed in patients without sputum production. Therefore, lung biopsy is often required to obtain histological evidence to rule out other diagnoses and to confirm the presence of TB, particularly in patients with unusual chest computed tomography (CT) findings, such as solitary pulmonary nodules or consolidation [7]. For lesions that are primarily located in the peripheral regions in chest CT or central lesions that cannot be directly observed by bronchoscopy, CT-guided percutaneous transthoracic needle biopsy (PTNB) is often needed to acquire lung lesion tissues [8].

Chronic granulomatous inflammation with central caseation is the characteristic histopathological finding in lung specimens from patients with TB; however, this observation alone is not sufficient for making a definite diagnosis. Although histological AFB (HAFB) staining of human tissue specimens is widely used to support the diagnosis of tuberculosis [7, 9–11], the sensitivity of HAFB is reported to be low (28–58%) for the diagnosis of PTB [7, 9, 11], and AFB staining cannot distinguish nontuberculous mycobacterium from tuberculosis mycobacterium. However, the specificity of HAFB is reported to be as high as 94% [7].

Due to the relatively high sensitivity, rapid reporting (1 or more weeks earlier than culture), lack of requirement for biosafety level 3 facilities, and relative ease of automation [12, 13], polymerase chain reaction (PCR) is becoming an increasingly important molecular diagnostic method for diagnosing mycobacterium tuberculosis infection using sputum, body fluids, and tissues [7, 10, 14–20]. However, only a few studies have used lung tissue specimens for evaluation, and all of the participants in these studies were suspected to have PTB [7, 19, 20]. Moreover, until now, no studies had reported the combination of AFB staining and TB-PCR in lung tissue specimens for the diagnosis of smear-negative PTB.

Therefore, in this study, we aimed to evaluate the clinical usefulness of the combination of HAFB staining and TB-PCR on lung biopsy specimens for the diagnosis of smear-negative PTB.

## Methods

### Study design and participants

This study was approved by the Institutional Review Board (IRB) of West China Hospital, and informed consent was waived. We included in-patients with suspected tuberculosis in West China Hospital Sichuan University from May 2012 to July 2015 who underwent CT-guided percutaneous lung biopsy and had AFB smear-negative sputum. PTNB was performed by two experienced physicians (3 years of experience with CT-guided PTNB), together with a technician and a nurse. Either automatic (Mn1816, C1816B; BARD, NJ, USA) or semiautomatic (QCS-18-15-20T; Cook, Bloomington, IN, USA) coaxial cutting needles were used. The PTNB method used in this study was reported in detail in our previous work (8). All lung biopsy specimens were fixed in formalin, embedded in paraffin, and subjected to HE staining, AFB staining, and TB-PCR. Clinical data, including sex, age, symptoms, CT findings, final diagnosis, and human immunodeficiency virus (HIV) status, were collected. All authors had access to information that could identify individual participants during and after data collection.

### Diagnosis of smear-negative PTB

The diagnostic criteria for smear-negative PTB were as follows: 1) definite TB [21], i.e., having sputum that was smear-negative for AFB on at least two sputum specimens at the start of treatment but culture-positive for *M*. *tuberculosis*; 2) probable TB [7], i.e., negative mycobacterial cultures from respiratory specimens but histopathological findings suggesting TB (chronic granulomatous inflammation with caseation necrosis, chronic inflammation, granulomas, or necrosis alone) and good clinical response to TB medication. Patients with other pathologically confirmed lung diseases (fungi, lung cancer, vasculitis, etc.) were enrolled in the non-TB group.

### AFB smears of respiratory and lung tissue specimens

Suspicious respiratory specimens (0.05 mL) were collected and spread evenly on slides to form a 10 mm × 20 mm sputum membrane. The specimens on the slides were then air dried, heat fixed, and stained using the Ziehl-Neelsen method. The lung tissues were fixed in 4% formalin and embedded in paraffin. In order to detect AFB, 8-μm-thick tissue sections were cut from each paraffin block, deparaffinized using xylene and100% ethanol, and stained using the Ziehl-Neelsen method. After conventional dehydration and mounting, the processed specimens were reviewed under oil immersion by two pathologists.

### Mycobacterial culture

A standard NALC-NaOH digestion-decontamination method [22] was used to process the respiratory specimens. The processed specimens were concentrated by centrifugation at 3500 rpm for 15 min. The concentrated specimens were then inoculated in the BACTEC Mycobacteria Growth Indicator Tube System (BACTEC 960/MGIT; Becton Dickinson Co., USA), which could automatically detect the fluorescence signal of O_2_ consumption caused by *M*. *tuberculosis* growth.

### DNA extraction of lung tissue specimens

TB-DNA from lung tissue specimens was extracted using an AmoyDx FFPE DNA Kit (Spin Column; Amoy Diagnostics Co. Ltd., Xiamen, China) according to the manufacturer’s instructions. The samples were resuspended in 180 μL buffer mixed with 20 μL proteinase K solution and digested at 56°C for 1 h. To inactivate proteinase K, the samples were mixed with 10 μL Buffer DES and incubated at 90°C for 1 h. Next, 200 μL Buffer DTB together with 200 μL of 100% ethanol was added to the samples, and samples were vortexed and centrifuged at 8000 rpm for 10 s. The supernatant was transferred to a DNA Spin Column. After two consecutive centrifugations at 8000 rpm for 1 min with the addition of 600 μL Buffer DW1 or Buffer DW2, respectively, the columns were transferred to new clean collection tubes and centrifuged at 14000 rpm for 3 min. The collection tubes were then discarded, and the columns were placed in new clean 1.5-mL centrifuge tubes. After adding 30–100 μL Buffer DTE to the center of the membranes, the tubes were incubated at room temperature (15–25°C) for 1 min. The samples were then centrifuged again (14000 rpm, 3 min) to elute the pure, concentrated TB-DNA.

### Reverse transcription polymerase chain reaction (RT-PCR) of IS6110 from *M*. *tuberculosis* in lung tissue specimens

PCR procedure was performed with a Care TB Diagnostic Kit for Mycobacterium Tuberculosis DNA (PCR-fluorescent probing; Qiagen China [Shenzhen] Co. Ltd., Shenzhen, China; S1 File) using a LightCycler 480 Real-Time PCR System (Roche Diagnostics, Germany). Uracil N-glycosylase enzyme (UNG) was used to control carry-over contamination in PCR [23]. PCR was carried out in a reaction volume of 20 μL, containing 17.8 μL master mix, 0.2 μL Taq DNA polymerase, 0.03 μL UNG enzyme, and 2 μL template DNA. The cycling conditions were as follows: antipollution at 37°C for 5 min, initial denaturation at 93°C for 1 min, and amplification by 40 cycles at 93°C for 5 s and 60°C for 40 s. Quality control and data analysis were performed according to the manufacturer’s instructions.

### RT-PCR and sequencing of *16S rRNA* for identification of *Mycobacterium* species in HAFB-positive and TB-PCR-negative specimens

The sequence 16S rRNA was amplified via real-time PCR, carried out using a Crystal Core *Mycobacterium* Species Identification Chip Kit (CapitalBio, Beijing, China) according to the manufacturer’s instructions. Different fluorescently labeled probes were designed according to the 16S rRNA sequences. The 20-μL reaction mixture contained 2 μL template DNA, 10 μL of 10× LA PCR buffer, 0.8 μL primer 16S rRNA-P1 (AGGTGGCTCAGGACGAACG; 10 μM), 0.8 μL primer 16S rRNA-P2 (AGCCGTGAGATTACACGCACA; 10 μM), 2.5 mM dNTPs, 1.6 μL, 0.25 μL of 5 U/μL Taq polymerase, and 12.55 μL ddH_2_O. Amplification was performed with an initial 10 min denaturation at 94°C, followed by 35 cycles of 30 s at 94°C, 30 s at 65°C, and 40 s at 72°C, with a final extension for 7 min at 72°C. The amplification products were then hybridized with chip probes under certain conditions. *Myobacterium* species could be identified according to the locations of specific probes on the chip.

To confirm the results of probe hybridization, 16S rRNA sequencing was carried out using an ABI Prism BigDye Terminator Cycle kit and an ABI Prism 3730XL instrument, with a BLAST search in GenBank.

### Statistical methods

The sensitivity, specificity, positive predictive value (PPV), negative predictive value (NPV), and accuracy for the diagnosis of smear-negative PTB were calculated for HAFB staining, RT-PCR for *M*. *tuberculosis*, and serial and parallel tests of HAFB staining and RT-PCR. Additionally, 95% confidence intervals (CIs) were estimated according to the binomial distribution. Chi-squared tests were used to compare the positive proportions, and results with *p* values of less than 0.05 were considered statistically significant. Analyses were performed using PASW Statistics 18.0 (SPSS; Chicago, IL, USA).

## Results

### Demographic and clinical characteristics of participants

A total of 236 patients were enrolled in this study; of these patients, 16 patients were excluded because of negative mycobacterial culture results and loss to follow-up. Another 39 indeterminate patients could not be ascribed to the TB or non-TB group according to the diagnostic criteria described above. After excluding these patients, a total of 181 patients were included in the analysis; of these, 133 were diagnosed with TB (nine confirmed TB, 124 probable TB), and another 48 patients received a final diagnosis of non-TB (Table 1).

**Table 1:** Baseline characteristics of the study patients.

Patient characteristics	TB	Non-TB	Total
**Patients n**	133	48	181
**Sex**			
Male	76	27	103
Female	57	21	78
**Age (years)**	51 (17, 80)	50 (23, 72)	
**Chest radiology findings**			
Typical	16	2	18
Atypical	117	46	163
**CT findings**^#^			
Nodule	57	3	60
Mass	39	8	47
Consolidation	14	2	16
Cavity	7	10	17
Other	16	25	41
**Sputum culture**			
Positive	9		
Negative	24		
**IGRAs (n)**			
Positive	112		
Negative	21		
**Final diagnosis**			
Malignancy		6	
Mycosis		8	
Nonspecific inflammatory lesion		25	
Other		9	
HIV positive*	0		

Data are presented as n (%) or median (range). CT: computed tomography; TB: tuberculosis; IGRA: interferon-γ release assay.

^#^ typical chest radiographic findings were defined as multiple nodular or interstitial infiltrates predominantly in the upper lobes;

* all patients received HIV tests, and none showed positive results.

### Diagnostic performance of HAFB and real-time PCR for analysis of lung biopsy specimens

The overall diagnostic performance of HAFB and real-time PCR for analysis of lung biopsy specimens for smear-negative tuberculosis is shown in Table 2. The sensitivity, specificity, PPV, NPV, and accuracy of HAFB were 61.7%, 100%, 100%, 48.5%, and 71.8%, respectively. Compared with HAFB, real-time PCR for *M*. *tuberculosis* showed relatively higher sensitivity, NPV, and accuracy (89.5%, 76.7%, and 91.2%, respectively) and similar specificity and PPV (98.5% and 98.3%, respectively). TB-PCR yielded two cases with false-positive results; one case was proven to have pulmonary aspergillosis, and the other case was proven to have a cryptococcal infection. Despite the positive PCR results, anti-TB medications were not used in the two patients because the clinical courses were more compatible with fungal infection than with TB.

**Table 2:** Diagnostic performance of HAFB and real-time PCR for analysis of lung biopsy specimens.

	TB (n = 133)	Non-TB (n = 48)	Total (n = 181)	Sensitivity%	Specificity%	PPV %	NPV %	Accuracy%
**AFB**								
Positive	82	0	82					71.8
Negative	51	48	99	61.7	100	100	48.5	
Total	133	48	181	(58.6–61.7)	(91.5–100)	(95–100)	(44.4–48.5)	
**PCR**								
Positive	119	2	121					91.2
Negative	14	46	60	89.5	95.8	98.3	76.7	
Total	133	48	181	(86.1–90.7)	(86.5–99.3)	(94.6–99.7)	(69.2–79.4)	
**PCR in HAFB negative patients**								
Positive	43	2	45					89.9
Negative	8	46	54	84.3	95.8	95.6	85.2	
Total	51	48	99	(76.3–87.5)	(87.3–99.2)	(86.4–99.2)	(77.6–88.2)	
**HAFB+PCR serial test**								
Positive	76	0	76					68.5
Negative	57	48	105	57.1	100	100	45.7	
Total	133	48	181	(54.1–57.1)	(91.5–100)	(94.6–100)	(41.8–45.7)	
**HAFB+PCR parallel test**								
Positive	125	2	127					94.5
Negative	8	46	54	94	95.8	98.4	85.2	
Total	133	48	181	(90.8–95.2)	(87.0–99.2)	(95.1–99.7)	(77.3–88.2)	

Fifty-one patients with smear-negative PTB also had negative HAFB results on lung biopsy specimens. The sensitivity, specificity, PPV, NPV, and accuracy of real-time PCR for HAFB-negative patients were 84.3%, 95.8%, 95.6%, 85.2%, and 89.9%, respectively. Compared with the results in HAFB-negative patients, RT-PCR showed relative better diagnostic performance for another 82 HAFB-positive patients, with sensitivity, specificity, PPV, NPV, and accuracy percentages of 92.7%, 95.8%, 97.4%, 88.5%, and 93.8%, respectively.

Serial and parallel tests of HAFB and TB-PCR for the diagnosis of smear-negative PTB were also performed. A serial test of HAFB and real-time PCR showed that the sensitivity, specificity, PPV, NPV, and accuracy of this combined approach were 57.1%, 100%, 100%, 45.7%, and 68.5%, respectively. In contrast, parallel tests of HAFB and real-time PCR showed higher sensitivity, NPV, and accuracy (84.3%, 85.2%, and 94.5%, respectively) and similar specificity and PPV (95.8% and 98.4%, respectively).

Moreover, we also analyzed the time from the initial evaluation to the final report of mycobacterial cultures and HAFB/PCR results. The time from the first evaluation to the report of HAFB/PCR results was 12.3 ±14.5 (mean ± standard deviation [SD]) days, compared with 56.8 ± 28.2 days for mycobacterial culture. The combination of HAFB and TB-PCR provided an earlier diagnosis than mycobacterial cultures.

### Identification of *Mycobacterium* species in HAFB-positive and TB-PCR-negative specimens

A total of 10 cases were HAFB positive and TB-PCR negative on lung biopsy specimens (Table 3). After *Mycobacterium* species identification by *16S rRNA* PCR, specific probe hybridization, and sequencing (S2–S5 Files), seven cases remained PCR negative, of which four were clinically diagnosed as PTB, and three remained indeterminate. One case was proven to be *M*. *tuberculosis*, with positive TB-PCR results, and the remaining two cases were non-TB *Mycobacterium* species (NTM: one *M*. *avium*, one *M*. *kansasii*). The patient with *M*. *avium* infection was a 35-year-old man; chest imaging showed a mass on the left upper lobe. The patient with *M*. *kansasii* infection was a 20-year-old woman presenting with right side pleural effusion. Interferon-γ release assays (IGRAs) were negative for both NTM patients. All five patients with PTB were treated with first-line chemotherapy regimens. Of the three indeterminate patients, two were treated with first-line anti-TB regimens, and one was treated with regular antibiotics (piperacillin/tazobactam). The patient with *M*. *avium* infection was switched to a second-line chemotherapy regimen (pasiniazide, moxifloxacin, protionamide, and amikacin) due to lack of efficacy of the first-line anti-TB treatment. The patient with *M*. *kansasii* infection was still followed up with a first-line anti-TB chemotherapy regimen (isoniazid, rifapentine, ethambutol, and levofloxacin), which was also effective in the treatment of *M*. *kansasii* infection.

**Table 3:** Clinical characteristics of the 10 HAFB-positive and TB-PCR-negative patients.

No.	Sex	Age (years)	Final diagnosis	Radiology findings	IGRAs	Treatment	16S rRNA PCR
1	F	59	Tuberculosis	Plaque and consolidation	+	First-line anti-TB regimen	-
2	F	29	Tuberculosis	Nodules	+	First-line anti-TB regimen	-
3	M	74	Indeterminate	Plaque and consolidation	-	First-line anti-TB regimen	-
4	M	35	***M*. *avium***	Mass	-	Second-line anti-TB regimen	+
5	F	60	Tuberculosis	Plaque and consolidation	+	First-line anti-TB regimen	+
6	M	51	Indeterminate	Nodules	+	Antibacterial treatment	-
7	F	52	Tuberculosis	Mass	+	First-line anti-TB regimen	-
8	M	42	Indeterminate	Nodules	+	First-line anti-TB regimen	-
9	F	70	Tuberculosis	Mass	+	First-line anti-TB regimen	-
10	F	20	***M*. *kansasii***	Hydrothorax	-	First-line anti-TB regimen	+

F, female; M, male; +, positive; -, negative; IGRAs, interferon-γ release assays.

## Discussion

Many previous studies have reported the diagnostic performance of HAFB staining and various types of PCR using formalin-fixed, paraffin-embedded human tissues for the diagnosis of tuberculosis [7, 9–11, 18, 20, 24, 25]. Among these studies, only two have reported the diagnostic performance of HAFB and PCR for PTB specifically in formalin-fixed paraffin-embedded lung tissue specimens [7, 20], and one of these two studies had a small sample size of only 25 individuals [20]. Although the other study included a greater number of specimens and reported comparable diagnostic performance of both HAFB (sensitivity: 58% versus 61.7%; specificity: 94% versus 100%, respectively) and PCR (sensitivity: 85% versus 89.5%; specificity: 99% versus 95.8%, respectively) relative to the result of our study [7], the case group included patients with PTB rather than only patients with smear-negative PTB.

In this study, we evaluated the combination of HAFB and RT-PCR specifically for the diagnosis of smear-negative PTB using formalin-fixed paraffin-embedded lung tissue specimens. Our findings suggested that, compared with HAFB staining of lung tissue specimens, RT-PCR showed comparable specificity (95.8% versus 100%, respectively) and higher sensitivity (89.5% versus 61.7%, respectively), consistent with our previous studies [7, 20]. For HAFB-negative patients, RT-PCR still exhibited considerable diagnostic yield in our study, which has not been reported in other studies. Serial RT-PCR and HAFB tests did not improve the diagnostic specificity significantly (from 95.8% to 100%) and showed lower sensitivity (52.03%). Moreover, parallel RT-PCR and HAFB tests can improve the sensitivity (94%) without obvious decreases in specificity (95.8%). Thus, for the diagnosis of smear-negative PTB, parallel RT-PCR and HAFB tests had relatively high diagnostic yield because of the high sensitivity (94%), specificity (95.8%), and accuracy (94.5%). In conclusion, for patients with suspected smear-negative PTB with no obvious TB-related symptoms, nonspecific CT findings, lung lesions that may be difficult to observe directly by bronchoscopy, or negative bronchoscopy biopsy results, HAFB and TB-PCR results from CT-guided PTNB lung tissues are both recommended to obtain sufficient evidence to confirm the diagnosis of TB or rule out other diseases. Isolation of NTM from clinical pulmonary specimens has been increasing worldwide, with an average annual increase of 8.2–33.3% for the past two decades [26–33]. In China, an epidemiological survey of NTM infection from Shanghai showed that the isolate rate increased from 3.0% in 2008 to 8.5% in 2012, with *M*. *kansasii* accounting for the highest proportion (45.0%), followed by *M*. *avium* (20.8%) and *M*. *chelonae/abscessus* (14.9%) [31]. In our study, after identification of *Mycobacterium* species in 10 HAFB-positive and TB-PCR-negative specimens by real-time PCR and sequencing of 16S rRNA, two were proven to be NTM, and the clinical courses of both cases were consistent with NTM infection. One other patient had a second TB-PCR-positive result, which could be explained by different amplification sequences or inappropriate or insufficient amounts of tissue specimens for the first PCR. *M*. *leprae* has nearly been eliminated in China; thus, the other seven HAFB-positive patients with two negative PCR results may be explained by inappropriate or insufficient amounts of lung tissue specimens or DNA degradation due to long-term storage of lung tissues (more than 1 year in our study). Therefore, it may be important to obtain appropriate and sufficient amounts of lung tissue specimens and perform immediate PCR of specific fragments of DNA/RNA in lung tissue specimens to increase the diagnostic sensitivity. Additionally, for patients with HAFB-positive and TB-PCR-negative results, it is necessary to perform analysis for identification of *Mycobacterium* species by hybridization and/or sequencing of specific fragments of mycobacterial genomic DNA using specific probes in order to avoid unnecessary anti-TB treatment.

For patients positive for both HAFB and TB-PCR, evidence may be sufficient for initiation of anti-TB treatment. However, for HAFB-positive and TB-PCR-negative patients, NTM infection cannot be ruled out. In our study, a second PCR of 16S rRNA and specific probe hybridization were performed to identify potential NTM. Although biopsy tissues were stored for more than 1 year before the second PCR, two of 10 were still proven to be NTM by probe assays, and the results were confirmed by sequencing; another sample was proven to be TB. RT-PCR may cost more than conventional PCR; however, this method allows for automatic detection of specific fragments of DNA, and overall cost savings can be achieved using the results for prioritizing contact investigations, making decisions regarding the results, or reducing nonindicated TB treatment [6, 34]. This study also had several limitations. First, we did not use an identical protocol for determining whether to perform CT-guided lung biopsy because this was a retrospective study. Second, mycobacteria cultures were not performed for all lung tissues or respiratory specimens. Therefore, only nine patients with PTB were bacterially confirmed; this may have influenced the power of the diagnostic tests in our study. Third, species identifications were only performed on patients with HAFB-positive and TB-PCR negative results; there were still seven HAFB-positive patients whose *Mycobacterium* species could not been determined because of a second negative 16S rRNA PCR result.

## Conclusion

Parallel HAFB staining and TB-PCR tests led to high diagnostic yields in patients with smear-negative PTB. For patients with HAFB-positive and TB-PCR-negative results, NTM infection should be suspected, and species identification is necessary using specific probe hybridization or sequencing of specific fragments of the mycobacterial DNA/RNA.

## Supporting Information

S1 File Care TB 1.0 handbook. (PDF)

S2 File 16S rRNA sequence of the M. kansasii specimen. (DOC)

S3 File 16S rRNA sequence of the TB specimen. (DOC)

S4 File A Set of Minimal Data of the Study. (XLSX)

S5 File English Version of Approval from IRB of West China Hospital. (DOC)

S1 Fig 16S rRNA sequence diagram of the M. kansasii specimen. (PDF)

S2 Fig 16S rRNA sequence diagram of the TB specimen. (PDF)

S3 Fig Original of Approval from IRB of West China Hospital. (JPG)
