# Supplementary material for: Nucleic Acid Amplification Testing and Sequencing Combined with Acid-Fast Staining in Needle Biopsy Lung Tissues for the Diagnosis of Smear-Negative Pulmonary Tuberculosis
Source: PLoS One. 2016 Dec 2;11(12):e0167342. doi: 10.1371/journal.pone.0167342 (PMC5135092; doi:10.1371/journal.pone.0167342)
Supplement: S1 File — (PDF) [file pone.0167342.s001.pdf]

QIAGEN

# *care*TB PCR ASSAY

# Instruction

**Cat. No. 4831014 / 4831024**

## 【Product Name】

Brand Name: *care*TB PCR ASSAY

English Name: Diagnostic Kit for Detection of Mycobacterium Tuberculosis (TB) DNA（real-time PCR）

## 【Packing Specification】

48 tests/ kit; 96 tests/kit

## 【Intended Use 】

Mycobacterium Tuberculosis (TB) is the pathogenic bacteria that cause tuberculosis. It could infringe each organ of the whole body, but the most common is tuberculosis. As living standard and health condition improve, especially the mass prevention treatment, especially after children generally receive BCG vaccine, the morbidity and mortality of tuberculosis (TB) is greatly reduced. However, due to the HIV/AIDS and the emergence of drug-resistant strains of mycobacterium tuberculosis, the application of immunosuppressant, drug abuse, poverty and population mobility, the TB epidemic has a rising trend.

This kit could be used for qualitative detection of tuberculosis with tubercle bacilli sample taken from suspected patients; it could assist tuberculosis diagnosis.

## 【Principles】

This kit uses a pair of PCR primers and a dual-labeled fluorescence probe, which could combine the DNA template during the primer amplification. Through PCR technology and fluorescence detection technology combination, automatically detection of TB DNA could be realized. After the circulation completes, there is no need to open the cover. Also the kit uses dUTP-UNG to avoid contamination.

## 【Kit contents】

| Diagnostic Kit for Mycobacterium Tuberculosis (TB) DNA（PCR-Fluorescent Probing） | **I**<br>（**20 μL system**）<br>**1061102** | **II**<br>（**40 μL system**）<br>**1061085** |
|---|---|---|
| **Sample Preparation Reagent** | | |
| TB DNA Extraction Solution | 1 mL×2 tube | 1 mL×2 tube |
| **TB PCR Solution** | | |
| TB PCR Solution | 1 mL×1 tube | 1 mL×2 tube |
| Taq DNA polymerase（5 U/μL） | 10 μL×1 tube | 10 μL×1 tube |
| UNG（1 U/μL） | 5 μL×1 tube | 5 μL×1 tube |
| **Controls** | | |
| TB Negative Control | 1 mL×1 tube | 1 mL×1 tube |
| TB High Positive Control | 1 mL×1 tube | 1 mL×1 tube |
| TB Critical Positive Control | 1 mL×1 tube | 1 mL×1 tube |

Note: each component of the 96 tests/kit is twice as above except that the control amount is the same.

Materials needed but not provided: 4% NaOH, sterile normal saline

## 【Kit storage and lifetime】

The kit should be stored at -20℃ with lifetime as 12 months (please use before the Expiry Date)

## 【Applicable platforms】

iCycler, ABI 5700/7700/7000, LightCycler, Rotor-Gene Q, Opticon Monitor, Line-Gene.

## 【Requirement of Samples】

1. Sample collection: better to use the first sputum in the early morning. The patient should first use clean water to blush the teeth, and then cough up the sputum and store in the sterile sample tube, seal it and send for test.
2. Storage: sample should be stored between 2℃ to 8℃ for no more than 24 hours, stored at -20℃ for no more than 3 months, stored at -70℃ for a long time. The sample should avoid repeated freezing and thawing of sample.
3. Transport: Transport the specimen(s) in sealed containers packed on ice (or equivalent) in and insulated transport container.

## 【Protocol】

**1. Specimen Preparation (performed in Specimen Preparation Area)**

1.1. First, visually judge the sputum, if saliva is the majority in the sample, need to re-collect the sample.

1.2. If the sample is qualified, add 2 to 3 times of 4% NaOH solution and place it on the shaker, treat it at 150 rpm、37°C for 30 minutes or place at room temperature for 1 hour to make it fully liquefied.(if there is no obvious solid substance and without dragline when absorb out, it means the sample is completely liquefied; if it is not completely liquefied, add a small amount of 4% NaOH solution until it is liquefied entirely)

1.3. Add 900 μL of sample, 500 μL of High Positive Control, Critical Positive Control, Negative Control each into the 1.5 mL microcentrifuge tube respectively; Centrifuge at 13,000 rpm for 10 minutes.

1.4. Discard the supernatant (first pour out most of the solution, pipette out the rest until there is no obvious droplet), add 1 mL sterilized saline, mix by vortexing (note: tighten the micro centrifuge tube micro centrifuge tube cover, place the tube horizontally on the vortex shaker to vortex the sediment) Centrifuge at 13,000 rpm for 10 minutes.

1.5. Discard the supernatant, use 1 mL sterilized saline to wash the sediment, centrifuge at 13,000 rpm for 10 minutes.

1.6. Discard the supernatant, add 30 μL DNA Extraction Solution into the sediment, mix by vortexing, centrifuge at 2,000 rpm for 5 seconds, and next incubate at 37°C for 30 minutes, and then incubate it at 100°C for 10 minutes, centrifuge at 13,000 rpm for 10 minutes, and then keep the supernatant for further use. (In case not to use the lysate on that day, store it at -20°C.)

**2. Amplification reagent preparation (PCR preparation area )**

Take out the TB PCR PCR Solution, Taq DNA polymerase, UNG, thaw it at room temperature, mix and centrifuge at 2,000 rpm for 10 seconds.

Assume the needed RCR reaction tubes as n(n=sample amount + 1 Negative Control +1 High Positive Control +1 Critical Positive Control), each test reaction system is formulated as the following table：

| Reaction system | Florescence PCR instrument | TB PCR Solution | Taq DNA polymerase | UNG |
|---|---|---|---|---|
| 40 μL | iCycler、ABI 5700/7700/7000, Opticon Monitor, Line-Gene | 37.8 μL | 0.2 μL | 0.06 μL |
| 20 μL | LightCycler, Rotor-Gene Q | 17.8 μL | 0.2 μL | 0.03 μL |

Calculate the amount of each reagent. Add them into a proper volume tube, fully mixed, centrifuge at 2,000 rpm for 10 seconds; aliquot the mixture into n PCR reaction tubes (for 40 μL reaction system, add 38 μL , for 20 μL reaction system, add 18 μL ), transfer them to the sample preparation area.

**3. Sample Loading(Sample Preparation Area)**

In case the sample lysate is stored at-20°c, thaw at room temperature before use, and centrifuge at 13000 rpm for 5 minutes.

Add sample, Negative Control, High Positive Control and Critical Positive Control 2 μL each prepared at step 1 in each PCR tube respectively. Fasten the lid of PCR tube (when use Roche capillary, place the capillaries into the LightCycler Centrifuge

Adapters, centrifuge at 2000 rpm for 10 seconds). Transfer the PCR tubes to the PCR amplification instrument. Mark the sample order.

**4. PCR Amplification (Detection Area)**

4.1. Temperature profile of PCR is set as follows:

For ABI 5700/7700/7000 PCR instrument:

37$^o$c：5 min；94$^o$c：1 min；
95$^o$c：5 sec，60$^o$c：30 sec，
40 cycles, the reaction system volume is 40 µL;

For icycler:

37$^o$c：5 min；94$^o$c：1 min；
95$^o$c：5 sec，60$^o$c：30 sec，
42 cycles, the reaction system volume is 40 µL;

For LightCycler:

37$^o$c：3 min；93$^o$c：1 min；
93$^o$c：5sec，60$^o$c：40 sec，
40 cycles, the reaction system volume is 20 µL;

The program setup should be before the amplification, set the sample and controls as "UNKN".

4.2. Instrument Channel selection

For multi-channel fluorescence instrument, the signal is collected at channel Fam and 60℃.

For LightCycler, the signal is collected at channel F1 and 60℃., and collection method is SINGLE.

For other kinds of instrument, please refer to the above and the instrument instruction and the actual circumstance.

## 【Reference Value (Range) 】

**1. Result Analysis Setup**

1.1. When use ABI5700/7000, the baseline setup should choose fluorescence signal of 6～10 or 6～15 cycles. The threshold should be set just over the highest point of the normal Negative Control amplification curve. (Irregular noise line), and Ct value = 40.0

1.2. When use ABI7700, the baseline setup should choose fluorescence signal of 3~10 or 3～15 cycles. The threshold should be set just over the highest point of the normal Negative Control amplification curve. (Irregular noise line), and Ct value = 40.0

1.3. When use iCycler, the baseline setup should be choose fluorescence signal of 2~10 or 2～15 cycles. The threshold should be set just over the highest point of the normal Negative Control amplification curve. (Irregular noise line), and Ct value = 0.0. And the Ct value could also adjust between 15.0～40.0 according to the noise.

(When use the above instrument for analysis, in order to avoid leaving out the High Positive Control, first set the baseline as 6～10/3～10/2～10 cycles, observe the Ct value, if there is no sample with Ct value＜16.0, then adjust the baseline as 6～15/3～15/2～15 cycles for results analysis; if there is sample with Ct value＜16.0, then discard this sample from the result analysis, meanwhile, the baseline is adjusted as 6～15/3～15/2～15 cycles for analysis.

1.4. When use LightCycler, read the test result in channel F1. The threshold should be set just over the highest point of the normal Negative Control amplification curve. (Irregular noise line),and with no Ct value( normally the baseline is within 0.001～0.05, but could be adjusted according to the actual condition.)

1.5. For other kinds of PCR instrument, please refer to the above baseline and threshold setup principle and adjust according to the instruction and actual condition.

**2. Quality Control**

2.1 $C_T$ value of Negative Control should be 40.0 (ABI5700/7700/7000) or 0.0(iCycler)）or no value （LightCycler）.

2.2 CT value of High Positive Control should be: CT value＜25.0

2.3 CT value of Critical Positive Control should be: CT value＜35.0, while also the CT value of Critical Positive Control should be higher than CT value of the High Positive Control .

Otherwise, the test is invalid.

## 【Interpretation of Results】

1. When $C_T$ value is 40（ABI7700/5700/7000）, 0（iCycler） and no value（LightCycler）, the report result is negative.
2. When $C_T$ value≤37.0, the report result is positive.
3. When $C_T$ value is higher than 37.0, suggest testing it again. When re-test result $C_T$ value＜40【iCycler:$C_T$ value＝0 &LightCycler: no $C_T$ value, do not belong this scope】, the report result is positive, otherwise, it is negative.

## 【Limitations】

The kit is targeted for detection of the pathogen nucleic acid amplification. Though in the kit design, we has choose relatively conservative fragments for amplification and detection, as the pathogen genes may mutant, despite that the mutant probability of mutation conservative area selected is very small, it could not completely avoid this possibility in theory.

## 【Performance Characteristics】

Limit of detection: 10 TB bacteria /mL

## 【Warnings】

1. This kit is only for in vitro detection.
2. Please read through this instruction before use.
3. The lab should be partitioned:
   First area: PCR preparation area--amplification reagent preparation.
   Second area: sample preparation area—sample and controls preparation.
   Third area: detection area—PCR amplification and detection.
4. The specific instruments and equipments should not be used interchangeably to avoid cross-contamination. Clean the working table immediately after the experiment.
5. Thaw the reagents and centrifuge briefly before use.
6. For sample stored at -20℃, thaw it at room temperature and centrifuge briefly before use.
7. The micro centrifuge tube filled with PCR Solution should be transferred to sample preparation area after covered or mount in the compacting bag.
8. The sample should be fully added into the PCR Solution, should not have the sample adhered to the tube wall, and cover the tube tightly as soon as possible after sampling.
9. Remove the reaction tube out immediately after amplification completed, and seal in a special plastic bag, discard in the specified place.
10. Avoided bubbles when vialing the PCR Solution, check each reaction tube as if it is tightly covered before put it into the instrument, so as to avoid the instrument polluted by the fluorescent substance leakage.
11. The used tips should be put into the waste cylinder containing 1% sodium hypochlorite, and discard with other discarded items together after sterilization.
12. Working table and each item should be disinfected with 1% sodium hypochlorite, 75% alcohol or UV lamp regularly.
13. Suggested Micro centrifuge tube and Tips

| Name | Specification | Manufacturer | Cat. No. |
|---|---|---|---|
| Tips | 10 µL | US AXYGEN | TF-300 |
| | 200 µL | | TF-200 |
| | 1000 µL | | TF-1000 |
| Micro centrifuge tubes | 0.5 mL | | MCT-060-C |
| | 1.5 mL | | MCT-150-C |

14. Materials Needed

| PCR preparation area | Sample preparation area | Detection area |
|---|---|---|
| Centrifuge;<br>Shaker;<br>Fridge（4°C、-20°C）<br>Pipette: one set;<br>Microcentrifuge tube shelf;<br>Consumables: disposable gloves, tips, microcentrifuge tube and glassware;<br>ultraviolet lamp.<br><br>Special clothing and office supplies | Tabletop high speed centrifuge;<br>Shaker;<br>Fridge（4°C、-20°C）;<br>Pipette: one set;<br>Fume hood, ultraviolet lamp;<br>Microcentrifuge tube shelf;<br>Consumables: disposable gloves, tips, microcentrifuge tube and glassware;<br>Special clothing and office supplies. | PCR instrument;<br>Ultraviolet lamp;<br>Special clothing and office supplies. |

**[Manufacturer]: QIAGEN (Shenzhen) Co. Ltd.**

**Registered address:**

6&7F, R3-B, High Tech Industrial Park, Nanshan District, Shenzhen

**Manufacturer address:**

6F, R3-B, High Tech Industrial Park, Nanshan District, Shenzhen;

5F, Unit 2, Building No.25 high-tech industry workshop, high-tech Road, Nanshan district, Shenzhen

**Zip code:** 518057

**Tel:** +86-755-86366188 **Fax:** +86-755-86366288

**Website:** www.qiagen.com

STATEMENT: CARE is a registered trademark of COOPERATIVE FOR ASSISTANCE AND RELIEF EVERYWHERE, INC.("CARE") CARE and the members and affiliates of CARE International are not affiliated with QIAGEN and do not sponsor, endorse, support, participate in or control the development, manufacture, use or sale of any QIAGEN product.
