# Supplementary material for: Nucleic Acid Amplification Testing and Sequencing Combined with Acid-Fast Staining in Needle Biopsy Lung Tissues for the Diagnosis of Smear-Negative Pulmonary Tuberculosis
Source: PLoS One. 2016 Dec 2;11(12):e0167342. doi: 10.1371/journal.pone.0167342 (PMC5135092; doi:10.1371/journal.pone.0167342)
Supplement: S2 File — (DOC) [file pone.0167342.s002.doc]

AAAGCGGCGTGCTTACACATGCAAGTCGAACGGAAAGGTCTCTTCGGAGACACTCGAGTG

GCGAACGGGTGAGTAACACGTGGGCAATCTGCCCTGCACACCGGGATAAGCCTGGGAAAC

TGGGTCTAATACCGGATAGGACCACTTGGCGCATGCCTTGTGGTGGAAAGCTTTTGCGGT

GTGGGATGGGCCCGCGGCCTATCAGCTTGTTGGTGGGGTGACGGCCTACCAAGGCGACGA

CGGGTAGCCGGCCTGAGAGGGTGTCCGGCCACACTGGGACTGAGATACGGCCCAGACTCC

TACGGGAGGCAGCAGTGGGGAATATTGCACAATGGGCGCAAGCCTGATGCAGCGACGCCG

CGTGGGGGATGACGGCCTTCGGGTTGTAAACCTCTTTCACCATCGACGAAGGTCCGGGTT

CTCTCGGATTGACGGTAGGTGGAGAAGAAGCACCGGCCAACTACGTGCCAGCAGCCGCGG

TAATACGTAGGGTGCGAGCGTTGTCCGGAATTACTGGGCGTAAAGAGCTCGTAGGTGGTT

TGTCGCGTTGTTCGTAATTCCCCCCGGTTAAAAA
