# Supplementary material for: Nucleic Acid Amplification Testing and Sequencing Combined with Acid-Fast Staining in Needle Biopsy Lung Tissues for the Diagnosis of Smear-Negative Pulmonary Tuberculosis
Source: PLoS One. 2016 Dec 2;11(12):e0167342. doi: 10.1371/journal.pone.0167342 (PMC5135092; doi:10.1371/journal.pone.0167342)
Supplement: S5 File — (DOC) [file pone.0167342.s005.doc]

**Approval document of IRB of West China Hospital, Sichuan University**

2015 No.192

| Department：Respiratory and Critical Care Medicine | | Principal investigator and title：Wang Ye, attending physician | |
| --- | --- | --- | --- |
| Project title | Nucleic acid amplification testing and sequencing combined with acid-fast stain in needle biopsy lung tissues for the diagnosis of smear-negative pulmonary tuberculosis. | | |
| Research Protocol | Version No.: 1.1 | | Version date: 2015.9.30 |
| Informed consent | None | | |
| Review comments：   1. The principal investigator is qualified according to ethical requirements. 2. The research protocol conforms to ethical requirements. 3. Agree to waive informed consent.   Review result：◼ Approve 🞏 Approve after amendments 🞏 Needs amendments 🞏 Disapprove 🞏 cease or pause  Please obey relevant laws, regulations and rules (the SFDA's Drug Clinical Trial Quality Management Norms (2003), The Medical Instrument Clinical Trial Regulations (2004), the Ministry of Health’s Biomedical Ethical Review Rules For Research Involving Human (on trial) (2007)), and follow the protocol approved by ethics committee and informed consent to carry out clinical trials (research), so as to protect the interest and health of the subjects.  In the process of trial (research), if there is any change of the principal investigator or informed consent etc., please submit an application for amendment to review.  If any serious adverse event occurs, please submit the serious adverse event report timely; After an immediate report, please submit details of the follow-up report about the serious adverse event as soon as possible.  Please submit the annual and regular tracking reports. When anything happens that may significantly impact the trial (research) or increase the risk of the subjects, the applicant is obliged to submit a written report to the ethics committee timely.  If the trial (research) involves in subjects that do not conform to the inclusion criteria or conform to any exclusion criteria, or any subject was not informed to cease if he/she conformed to the termination criteria, or any subject receives the wrong treatment or wrong doses or prohibited drugs, or any other situations happens that go against the protocol or disobey ethical principles and norms that may lead to bad impact on the scientificity of the trial (research) or health/rights of subjects, the applicant /inspector / researcher should submit protocol deviation reports.  The applicant should submit suspend or terminate reports as the clinical trial (research) is suspended or terminated in advance. The applicant should submit concluding reports as the clinical trial (research) is completed.    Hospital (Seal): West China Hospital, Sichuan University  Chairman of committee (Signature): Zeng  2015/ 10/ 26 | | | |
