# Supplementary figures and images for: Nucleic Acid Amplification Testing and Sequencing Combined with Acid-Fast Staining in Needle Biopsy Lung Tissues for the Diagnosis of Smear-Negative Pulmonary Tuberculosis
Source: PLoS One. 2016 Dec 2;11(12):e0167342. doi: 10.1371/journal.pone.0167342 (PMC5135092; doi:10.1371/journal.pone.0167342)

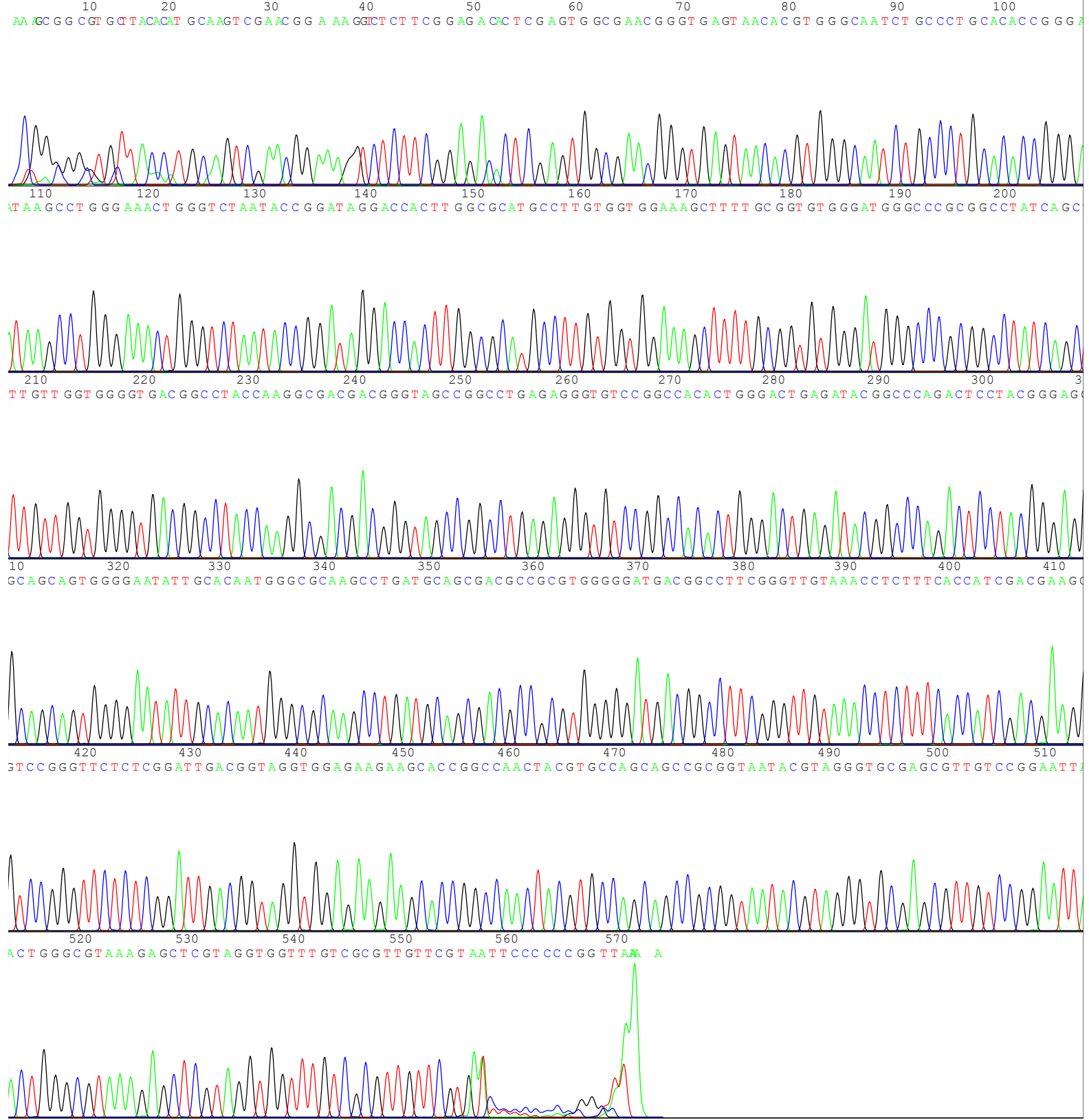

Supplement: S1 Fig — (PDF) [file pone.0167342.s006.pdf]

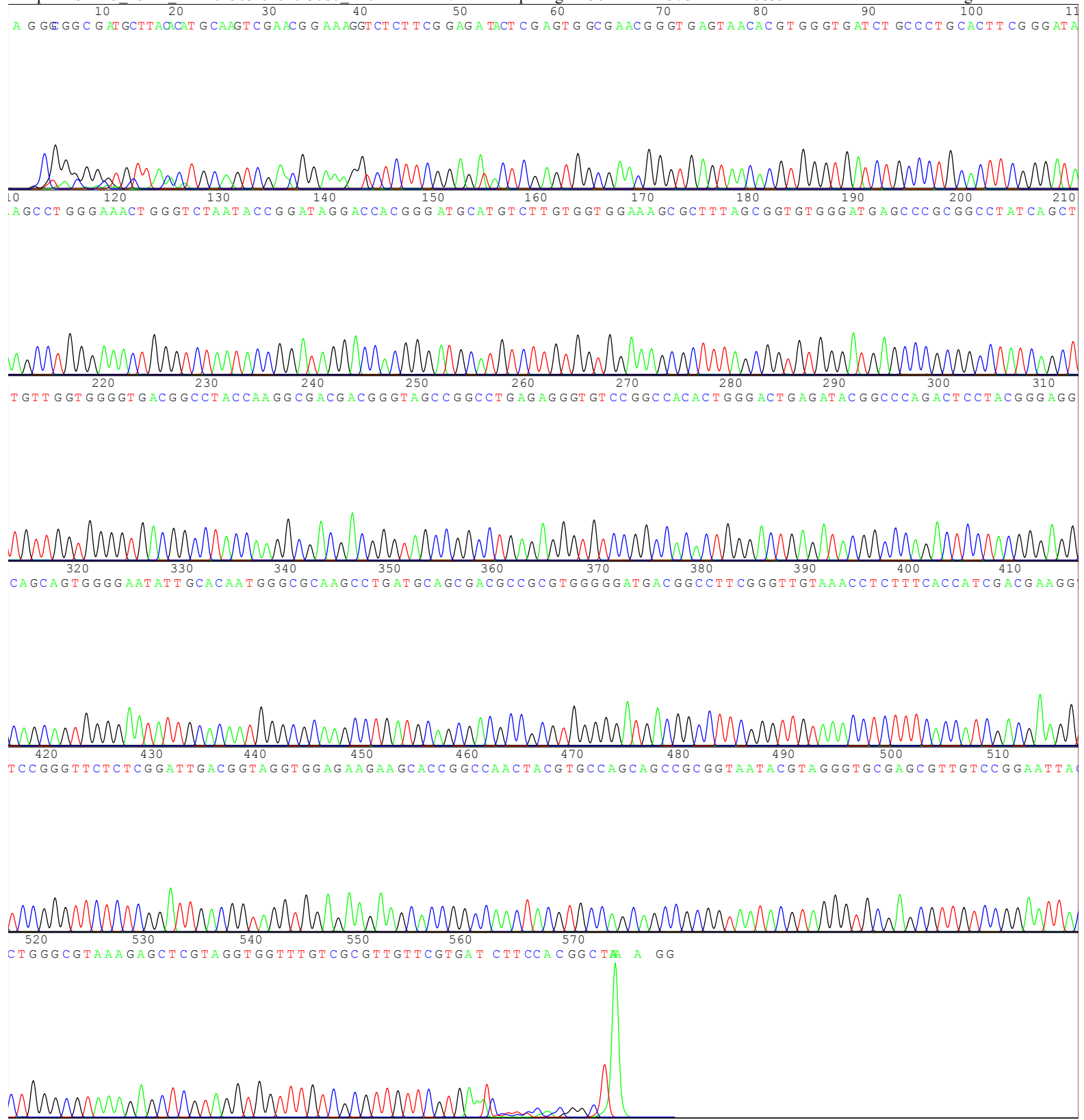

Supplement: S2 Fig — (PDF) [file pone.0167342.s007.pdf]

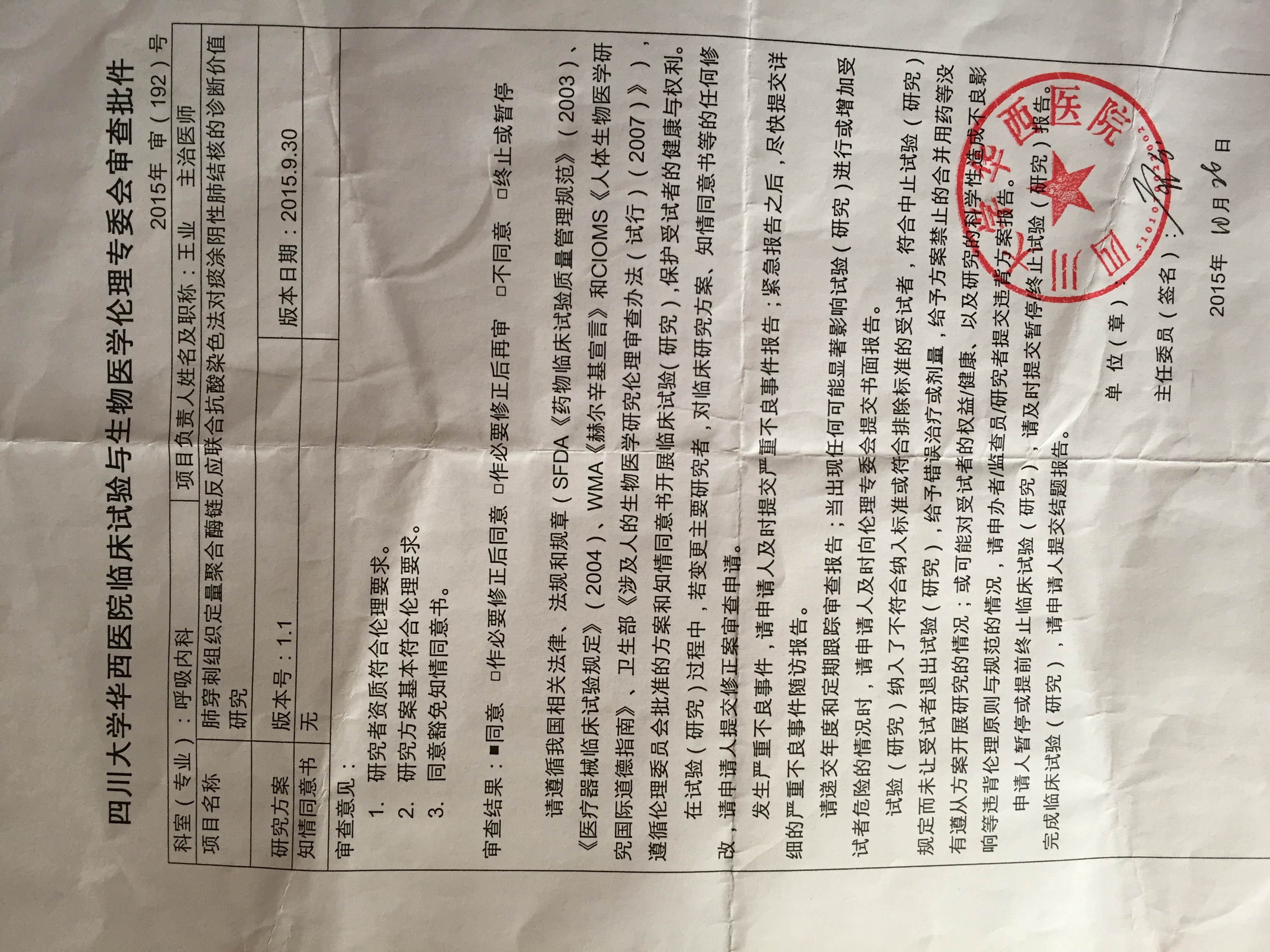

Supplement: S3 Fig — (JPG) [file pone.0167342.s008.JPG]
